# A Legal Framework to Support Development and Assessment of Digital Health Services

**DOI:** 10.2196/medinform.5401

**Published:** 2016-05-25

**Authors:** Cecilia Garell, Petra Svedberg, Jens M Nygren

**Affiliations:** ^1^ School of Health and Welfare Halmstad University Halmstad Sweden

**Keywords:** digital health, legal aspects, technological innovations

## Abstract

**Background:**

Digital health services empower people to track, manage, and improve their own health and quality of life while delivering a more personalized and precise health care, at a lower cost and with higher efficiency and availability. Essential for the use of digital health services is that the treatment of any personal data is compatible with the Patient Data Act, Personal Data Act, and other applicable privacy laws.

**Objective:**

The aim of this study was to develop a framework for legal challenges to support designers in development and assessment of digital health services.

**Methods:**

A purposive sampling, together with snowball recruitment, was used to identify stakeholders and information sources for organizing, extending, and prioritizing the different concepts, actors, and regulations in relation to digital health and health-promoting digital systems. The data were collected through structured interviewing and iteration, and 3 different cases were used for face validation of the framework.

**Results:**

A framework for assessing the legal challenges in developing digital health services (Legal Challenges in Digital Health [LCDH] Framework) was created and consists of 6 key questions to be used to evaluate a digital health service according to current legislation.

**Conclusions:**

Structured discussion about legal challenges in relation to health-promoting digital services can be enabled by a constructive framework to investigate, assess, and verify the digital service according to current legislation. The LCDH Framework developed in this study proposes such a framework and can be used in prospective evaluation of the relationship of a potential health-promoting digital service with the existing laws and regulations

## Introduction

Through the use of wireless devices, sensor technologies, the Internet, social networks, health information technology (IT), and personal health data, digital health services empower people to track, manage, and improve their own health and quality of life. At the same time, these services provide a more personalized and precise health care delivery, at a lower cost and with higher efficiency and availability [1]. An emerging area at the intersection of informatics, health care, and business is electronic health (eHealth) [2], which encompasses the mediation and interaction between health care and the individual via information and communication technology (ICT) [3]. Although the extent of implementation and application of eHealth systems vary, the overall goal is the same: using ICT to provide better care more efficiently at a lower cost [4]. Mobile health (mHealth), as a component of eHealth, involves the use and capitalization on mobile devices [5] and encompasses any use of mobile technology to address health care challenges such as access, quality, affordability, matching of resources, and behavioral norms [6]. The use of mHealth offers great opportunities by allowing asynchronous and remote care [7] to an extensive number of potential users [5]. Applications for mHealth serve a variety of functions: providing easy access to medical information about the symptoms and treatment of various diseases or allowing patients to track clinical measurements that can be sent to the care provider [6]. These applications could change the nature of health care [8] by using technology to increase patient engagement, improve care quality, transform care processes [6], reduce health care costs, and minimize human error [9].

Essential for the use of all digital health services is that the treatment of any personal data is compatible with the Patient Data Act, Personal Data Act, and other applicable privacy laws. The European Commission has declared its intention to drive greater legal certainty in the digital health domain, and through the Directive 2011/24/European Union (EU), for the first time, it has placed eHealth in a legal context, requiring member states to cooperate with interoperability standards to allow full use of eHealth services across EU borders [10]. Although some significant steps have been taken toward attaining this goal, the questions of liability for eHealth goods and services are still not fully addressed on EU level legislation. The lack of a fully worked out EU level framework illustrates the difficulties in pinpointing key concepts in relation to this rapidly evolving market. In response to this, the eHealth Authority was formed in Sweden in 2014 with responsibility for registries and the heterogeneity and variety of IT functions developed within Swedish health care.

While the authorities investigate and consider the technological capabilities of eHealth services in the intersection of health care quality, patient safety, ethics and legal matters, new IT services, and mobile applications are advancing dramatically. The focus for the regulatory authorities should be to streamline the regulatory processes and promote innovation [11], but because regulation and legislation are still behind, governmental authorities are forced to handle many issues in this domain case by case [10]. This implicates that designers of digital health services need to acquire knowledge about relevant regulation and legislation and how to relate to and act on such regulation [12]. A legal framework that could guide designers through these legal challenges, together with an understanding of the definitions of the concepts [13], would both simplify and speed up development of digital health solutions [14] and promote involvement of designers with experience from digital service design [15] in the development of new digital health services. The aim of this study was to develop such a framework to support designers in development and assessment of digital health services.

## Methods

The study design was based on a stakeholder analysis approach for generating knowledge about actors to understand their intentions, interrelations, and interests and for assessing their influence on legal challenges in development of digital health services [16]. Data obtained from interviews with relevant authorities and organizations together with information about concepts and regulations in relation to digital health services were analyzed and structured to create a framework for legal challenges.

### Case and Framing

A framing of the questions about legal challenges and key concepts relevant to development of digital health services was discussed in the project group and with a consulting firm (Carmona AB) with expertise in the field of Web-based services and information solutions for handling of patient data and quality control. The consulting firm is in the forefront of developing such services in accordance with current legislation and in development of new practices and legislation. In this communication, we used data from our development of a digital service for play and interaction between children, aged 8-12 years, who have survived from childhood cancer treatment to frame legal challenges and key concepts [17]. The case was described by a concept description [18] and use experience descriptions through Persona characters and use scenarios [19].

On the basis of this, a basic understanding of the domain was formed, and a major law firm, with experience of legal issues in health care and a jurisconsult responsible for privacy and patient safety issues at the county council, was consulted with the intention to extend knowledge and our preunderstanding of the legal challenges and key concepts in this domain. A first draft was conceived, of a legal framework with relevant concepts, laws, and agencies or organizations involved in the care of the target group, or with regulatory or supervisory responsibility.

### Information Sources

A purposive sampling [20] was used to identify stakeholders and information sources for organizing, extending, and prioritizing the different components of the framework guided by the case. The first contacted stakeholders referred to other stakeholders, that is, a snowball recruitment [21]. The information sources identified and used are listed in Table 1.

**Table 1:** Identified actors, organizations, and authorities, and their area of expertise, to be considered in the following investigation.

Actor	Area of expertise
The project group	Researchers focused on development of digital health services for children using a participatory design where researchers collaborate with children from the target group.
A local consulting firm	Specialized in development of Web-based services and information solutions
Data Inspection Authority	Works to secure the individual’s right to integrity in society
Inspection Authority for Health Care	Supervises the activities in the social area and health care, as well as of health care professionals; the Authority is also responsible for certain permits.
The National Board	Works for all citizens’ equal access to good health and health care
Ministry of Social Affairs	The different disciplines within the overall responsibility: health care, health, social issues, social security features news about the government’s policy initiatives or decisions; they also contain current objectives and the government’s priorities in the field.
County Council	Responsible for many aspects of development in the county; the County Council has the mission to promote development and growth and to provide good health care.
eHealth Authority	Works with the development of national eHealth to contribute to better health care and health; the business is focused on creating participation for residents and providing support to practitioners and policy makers.
European Commission	Represents interests of the EU^a^; the commission proposes new legislation to Parliament and the Council of Ministers and ensures that EU countries apply EU law correctly.
Medical Products Agency	Government agency under the Ministry of Social Affairs; it has the mandate to promote the Swedish public and animal health.

^a^EU: European Union.

### Data Collection

Identified websites of organizations, authorities and different operators or actors, and functions were screened for information about concepts and regulations in relation to digital health services. Stakeholders were interviewed about their relationship to eHealth and digital health services (Table 1). Interviewees were representatives from the County Council Board on Coordination of Information Safety, The National Board, The Data Inspection Authority, eHealth Authority, and Inspection Authority for Health Care. Interviews were performed, with 1 person from each of the aforementioned organizations, over phone (approximately 30 minutes) and repeated if new questions appeared. The topics in the semistructured interview guide were as follows: (1) Relationship to digital health services; (2) the authority’s function, assignment, and work for digital health services; (3) regulations that govern the work; and finally (4) other relevant information sources we should approach. In cases where we wanted to get the data confirmed in writing, follow-up questions were sent by email to the respective informant.

### Data Analysis

The meaning out of the data was made in a systematical way to discover the relevant concepts and relationships among the input [22]. All data inputs, such as questions, concept descriptions, laws and regulations, and functions, were put on post-it notes by the main author and structured on different levels and in relation to each other, and an affinity diagram was formed and discussed between all authors. The insights gained were used as a starting point for a framework for assessing the legal challenges in developing health-promoting digital services. The framework was iteratively verified against the project group and stakeholders (the Data Inspection Authority and eHealth Authority) and finally validated against three cases of digital health services.

## Results

### Identification of Concepts and Regulations

The identified concepts to consider in this domain are: medical device, eHealth, medical responsibility, care damage, personal data, and consent. The concepts, their definitions, and relevant regulations identified during data collection and the subsequent analysis are listed in Table 2. Concepts and regulations that were identified during data collection but were not found to be relevant for framing of legal challenges from the perspective of development of digital health services are not included in this compilation, such as: health care quality registries, the law on drug lists, and the regulations of The National Board of Health and Welfare.

**Table 2:** The Legal Challenges in Digital Health (LCDH) Framework for exploring a prospective health promoting digital service’s relationship to valid regulations.

#	Concept	Definition	Question	The following is valid for “yes”	The following is valid for “no”	Regulation
1	Medical device	A product is a medical device if it has a medical purpose as to: - Prove, prevent, monitor, treat, or mitigate a disease. - Prove, monitor, treat, mitigate, or compensate an injury or disability. - Examine, change, or replace anatomy or a physiological process. - Control fertilization.	Is the product a medical device?	The manufacturer must handle security aspects. Medical Products Agency is responsible for supervision of products and manufacturers. Inspection Authority for Health Care audits healthcare usage. *Proceed to No. 2.*	The manufacturer cannot claim anything, which is covered by the definition of a medical device, for example, that the product may mitigate a disease. *Proceed to No. 2.*	The law of medical devices (SFS^a^1993:584). Council Directive 93/42/EEC^b^concerning medical devices.
2	eHealth	An eHealth service has a purpose to: - Mediate health service or information and interaction between health care and an individual. - Mediate information exchange between patients and health care professionals, hospitals, and other professionals within health care and networks for health information and telemedicine. - Use ICT^c^to improve the preventive work, diagnoses, health care, monitoring, or administration.	Is the product an eHealth service?	*Proceed to No. 4.*	*Proceed to No. 3.*	The Health Care Act (SFS 1982:763).
3	Medical responsibility	Usually referred to health professionals' medical professional liability in the care and treatment of a patient and the medical responsibility in a comprehensive organizational plan.	Is the service recommended/supplied by the health care? The health care recommends a service if they encourage or call for usage. It is not enough to only inform that the service is available.	The health care vouches for the safety and security of the technology and that the risk of care damage is low. The service is examined and evaluated by a number of criteria. *Proceed to No. 4.*	The health care has no responsibility. *Proceed to No. 5.*	The Health Care Act (SFS 1982:763).
4	Care damage	A damage that could have been avoided if adequate arrangements were taken in contact with health care. If medical device or eHealth service: The risk of care damage is determined by the level of care, the vulnerability of the target group, and how the usage is being monitored or followed up by the health care.	Is there any risk of care damage?	If the service provides monitoring/data logs that register threshold values or personal controls to prevent care damage, the responsibility of the health care is restricted. If no monitoring, the health care is responsible for preventing formation of care damage. *Proceed to No. 5.*	The healthcare has no responsibility. *Proceed to No. 5.*	Patient Safety Act (SFS 2010:659).
5	Personal data	Definition personal data: All information that can directly or indirectly be assigned to a physical person who is alive. Definition handling of personal data: Every action or series of actions taken regarding personal data (automatically or not). For example, collection, registration, usage, storage, organization, processing, and distribution.	Are personal data handled?	*Proceed to No. 6.*	To completely stay out of Privacy Act, the outcome measures of the patients must be anonymized. The health care has no responsibility.	Privacy Act (SFS 1998:204). Patient Data Act (SFS 2008:355).
6	Consent	Consent is defined as any freely given specific and unambiguous expression by which the registered person, after receiving information, accepts handling of personal data relating to him or her.	Does the service lack user agreement? An agreement in which the purpose with the service, privacy, terms of use, responsibilities, and similar are regulated.	The responsibility of the health care should be investigated/examined.	A responsibility agreement signed by adult or parent/advocate may disclaim the health care from responsibility.	Privacy Act (SFS 1998:204).

^a^SFS: Swedish Code of Statutes

^b^EEC: European Economic Community

^c^ICT: information and communications technology

### Structure of Concepts and Regulations Into a Framework

On the basis of the identified concepts, regulations, and stakeholders, we designed a framework for assessing the legal challenges in developing digital health services (Legal Challenges in Digital Health [LCDH] Framework) consisting of 6 key questions to be used in prospective evaluation of the relationship of a digital health service to existing laws and regulations (Table 2). The questions are sequentially arranged so that affirmative responses gradually delineate which parts of the law apply to a certain digital health service. Negative responses to the same questions show which laws and regulations that each service is exempt from.

### Validation of the Framework

The accuracy and quality of the LCDH Framework were assessed by the Swedish Data Inspection Authority and eHealth Authority and, finally, by the consulting firm, the law firm, and the jurisconsult involved in the framing of the data collection. The reviewed and iteratively revised framework was confirmed to be in accordance with current regulation, law and practice, and experience of these stakeholders. Because the stakeholders, during data collection, did not identify additional stakeholders or sources of information than those already included in our dataset (which means that saturation was achieved), the quality assessment of our framework indicated that it was valid and in line with current law and practice.

To assess the usability, and hence the face validity, for using the framework for development and assessment of products and services, we applied the framework for evaluation of the legal challenges in 3 cases entailing development of digital health services. The questions in the framework (Table 2) were used to systematically evaluate and frame the legal challenges for the development and implementation of the digital services, *Give Me a Break*, *Sisom* and *DELTA* (Multimedia Appendix 1).

#### Is the Product a Medical Device?

A medical device is a product with a medical purpose; as to prove, prevent, monitor, treat or mitigate a disease, and to prove, monitor, treat, mitigate, or compensate an injury or disabilities (Table 2). The 3 digital services *Give Me a Break*, *Sisom*, and *DELTA*, were developed to facilitate child peer support, communication between children and their care providers, and adolescent’s participation in schools related to their health, respectively. None of the services has medical functions such as handling, treating, or preventing disease or illness and should therefore, according to the definitions outlined in Table 2 , not be considered as medical devices.

#### Is the Product an eHealth Service?

An eHealth service mediates health information or service or interaction between health care and the individual (Table 2). The system owner and system administrator of each of the 3 services, as well as the support and maintenance from the operation manager who is responsible for all data, will be independent from health care providers and schools. In one case though, *Sisom*, the services by the health care providers will be mediated through the digital service and information about the users' personal data will be shared with the health care providers. This service should therefore be considered as an eHealth service. The other 2 services, *Give Me a Break* and *DELTA*, do not mediate any communication of personal data or sensitive interaction at all between health care providers and users and should therefore not be considered as tools or services that use ICT to improve the preventive work, diagnoses, health-care monitoring, or administration and hence therefore not be defined as eHealth services.

#### Is the Service Recommended/Supplied by the Health Care?

Two of the services, *Sisom* and *DELTA*, are recommended and supplied by the health care services who therefore have medical responsibility for the usage of the services and any potential consequences of usage. This responsibility is independent of whether the services are to be considered as eHealth services. The other service, *Give Me a Break*, is neither part of regular treatment nor used to improve health care according to the definition of an eHealth service. It is neither recommended nor supplied by the health care, and there is therefore no medical responsibility for the activities or the consequences of the interaction on the service that can be imposed on the health care providers.

#### Is There Any Risk of Care Damage?

According to the definition in Table 2 , care damage is a damage that could have been avoided if adequate measures were taken by health care. The 2 services recommended and supplied by the health care, *Sisom* and *DELTA*, are not associated with medical treatment but involve sharing of potentially sensitive personal information. Although the risk of care damage is limited to sharing of personal information, this entails privacy risks for which the health care is responsible. To prevent this, there is no follow-up or surveillance system in the services that automatically transfers personal information or use data to the health care. To protect the users, the services has well-ordered procedures for registration and login. All information transfers are performed by web encryption technology, and professionally trained personnel monitor all real-time activities and use logs. Moreover, in *DELTA*, abuse or misconduct can be reported by the users to be handled by the involved school personnel. Both systems thus have significant infrastructure for monitoring safety and security of the users without interfering with their integrity. For the other service, *Give Me a Break*, the health care will not have any medical responsibility, as it neither has a medical purpose nor is seen as health care or treatment. Consequently, although problems can arise, there can be no care damage per se.

#### Are Personal Data/Personal Information Handled?

Personal data are handled in all the 3 services and in some cases, such information is of sensitive nature as it relates to health and is coupled to the users identity through a personal code number, name, or photo. In *Sisom*, health care handles sensitive personal data coupled to health and the users' identity. In *Give Me a break* and *DELTA*, the personal data are however not of sensitive nature (not coupled to sensitive information about the users) but deal with their identities and therefore still must be handled with care. In all the 3 services, the users provide all data added into and shared in the system, and the users are the sole owners of the information that they share. In *Give Me a Break*, the personal and shared user profile is stored but can be deleted by the users themselves if they decide to no longer make it available to others on the service. The provider of each of the 3 services has complete responsibility for all personal data stored or shared. This includes responsibility to: inform about the purpose and use of the service; not publish or share sensitive personal data, if applicable, regularly monitor posts to discover offensive personal data; and promptly remove any offensive personal data.

#### Does the Service Lack User Agreement?

At registration and the first logon to all the 3 services, the users and their parents must approve an agreement in which the purpose of the service is outlined. The user agreement regulates privacy issues, terms of use, and responsibilities. Specifically, they state to what extent and how the services are a part of the user’s health care. For *Give Me a Break*, the user agreement also states that all use takes place on the users' own initiative and under own responsibility.

## Discussion

The aim of this study was to develop a framework for legal challenges to support designers in development and assessment of digital health services. The LCDH Framework presented herein was created based on concepts and regulations identified through interviews with authority representatives, and a process of stakeholder review and iterative revision of the developed framework confirmed that it was in accordance with current regulation, legislation, and practice. Usability evaluation against real cases of digital health services revealed how the definitions in the framework feasibly guided identification of distinctive and appropriate regulation to be considered and legal challenges to relate to given the nature of each of the evaluated services.

The work of government regulation and legislation of digital health services have not so far kept pace with the digital development. Digital health services in various forms are under rapid development and are involving several stakeholders and actors. Game and app developers, for instance, with innovative ideas for digital health may experience obstacles in implementation of digital health services in the interface between health care and individuals [23]. One problem can in many cases be the indistinct legislation.

This slow and perhaps circumspect legislation under construction may cause difficulties to developers of digital health services to acquire knowledge about relevant regulation and how to relate to and act on the regulation. Implications of this can be: (1) inaccuracies due to misinterpretations and (2) omitted development of digital health services owing to complexity in understanding the regulations. It would be desirable in the future that this type of regulation and legislation would be prepared in cooperation between the authorities, the developers, and the health care experts [12]. However, until then, there is a need for a dynamic tool, a framework, guiding designers and developers through the legal challenges in development work in the digital health domain, together with an understanding of the definitions of the concepts [13]. This is important both to simplify and speed up development of digital health solutions [14] and to promote involvement of developers experienced in digital service design [15]. There is a need for approaching and proceeding with legal challenges adjacent health care in the design development to facilitate the forthcoming implementation.

The LCDH Framework presented in this article has the qualifications to be a useful tool in guiding designers and developers through the legal challenges in development work in the digital health domain. The framework: (1) considers the current regulation and legislation that apply in the EU; (2) presents the definitions of relevant legal concepts; (3) is verified by the Swedish Data Inspection Authority and eHealth Authority; and finally, (4) is easy to use. The framework merely aims to guide development by identifying legal dividing lines between different digital health services in their product design. It has no legal power to determine guidelines, and a jurisconsult may need to confirm the legal application in case of uncertainties. Although the concepts used in the framework are based on legislation in the EU, it can be used in other contexts to understand the legal challenges and the hierarchy of the various concepts governing legislation within the digital health domain.

### Strengths and Limitations

As with all methods and studies used in research, certain limitations apply. The interviews were performed with 1 person from each organization or authority over the phone. Performing the interviews over phone was convenient and time-saving, and if the informants had text material to share, it was sent by email. Important information sources and stakeholders can be identified by using snowball recruitment [21]; however, there is a risk that important informants are missed by this approach. In our study, it is likely that we through this approach identified relevant informants as both the Swedish Data Inspection Authority and the eHealth Authority verified our report. The mapping was performed during the spring and summer of 2014 in accordance with the regulations prevailing in Sweden. The definition of eHealth is however taken from the European Commission’s declaration of eHealth [3].

### Conclusions

Consideration toward ethical aspects is a requirement for both performing and publishing research in relation to health and human subjects. However, as long as such ethical aspects are taken into account, no requirements are placed on that, and research should also be aligned with legal challenges that are relevant to the context of the research.

Structured discussion about legal challenges in relation to health-promoting digital services can be enabled by a constructive framework to investigate, assess, and verify the digital service according to current legislation. The LCDH Framework developed in this study proposes such a framework and can be used in prospective evaluation of the relationship of a potential health-promoting digital service to the existing laws and regulations. However, legislation regarding eHealth in general and health-promoting digital services in particular is under construction, and authorities’ judgments are made from case to case. Further research is critical to expanding the knowledge base of cases, or products, using health-promoting digital service implemented and where current legislation is applied.

## Abbreviations

eHealth: Electronic health
EU: European Union
ICT: information and communications technology
LCDH Framework: Legal Challenges in Digital Health Framework
mHealth: mobile health

## Appendix

Multimedia Appendix 1 Usability validation of The Legal Challenges in Digital Health (LCDH) Framework for exploring the relationship to valid regulations of 3 health-promoting digital services.
