# Supplementary material for: A Legal Framework to Support Development and Assessment of Digital Health Services
Source: JMIR Med Inform. 2016 May 25;4(2):e17. doi: 10.2196/medinform.5401 (PMC4909384; doi:10.2196/medinform.5401)
Supplement: Multimedia Appendix 1 [file medinform_v4i2e17_app1.pdf]

Supplementary Table 1. Usability validation of The Legal Challenges in Digital Health (LCDH) Framework for exploring the relationship to valid regulations of 3 health-promoting digital services.

Health-promoting digital service					
No	Concept	Question	Sisom[a]	Give Me a Break[b]	DELTA[c]
1	Medical device	Is the product a medical device?	No	No	No
2	eHealth	Is the product an eHealth service?	Yes	No	No
3	Medical responsibility	Is the service recommended/supplied by the health care?	Yes	No	Yes
4	Care damage	Is there any risk of care damage?	Yes	No	Yes
5	Personal data	Are personal data handled?	Yes	Yes	Yes
6	Consent	Does the service lack user agreement?	No	No	No
Summary statement			Sisom is an eHealth service. Health care is	Give Me a Break is a digital service for	DELTA is a digital service. Health care is

		responsible for the medical purposes of the service and the potential risk of care damage. Personal data are handled and agreed on through a consent procedure.	which health care has no responsibility. Personal data are handled and agreed on through a consent procedure.	responsible for the medical purposes of the service and the potential risk of care damage. Personal data are handled and agreed on through a consent procedure.

[a]*Sisom* is a digital communication tool that assists children (6-12 years) in health care in communicating their experiences, feelings, life situation, and health. The child creates an avatar that sets out on a journey between islands on which the child finds a wide array of various experiences and symptoms represented by animations and brief statements to respond to. A summary of all responses is accumulated and eventually printed out as a hard copy that the child brings to the consultation with the medical staff. The use of *Sisom* results in a significant increase in symptoms and problems addressed and an improved quality and child focus of the health care dialogue.

[b]*Give Me a Break* is an interactive platform for play and social interaction, a safe meeting place where cancer survivors (8+ years) can interact with peers, find new friends, and build long-lasting friendships. The platform is composed of a virtual playground to connect users, creative playful activities facilitated by an online youth worker to stimulate interaction and integration of social media applications to precede interaction into other channels. The service is introduced at discharge from intensive care or during clinical checkups at the hospital after completion of treatment.

[c]*DELTA* is a digital service used for creating a greater sense of school ownership of adolescents and to support a sense of control and ability to manage school-related stress. The digital service supports a new approach for the school health team based on continuous interaction between the team and the school children. The children report daily on an app about how they feel being in school, and this information is summarized and returned to the school health team on a weekly basis for structured reflection, response, and feedback.
